# Time to integrate “One Health Approach” into nanoplastic research

**DOI:** 10.1016/j.eehl.2023.01.001

**Published:** 2023-01-21

**Authors:** Mohamed Helal, Nanna B. Hartmann, Farhan R. Khan, Elvis Genbo Xu

**Affiliations:** a Department of Biology, University of Southern Denmark, Odense, 5230, Denmark; b Department of Environmental and Resource Engineering, Technical University of Denmark, 2800 Kgs. Lyngby, Denmark; c Norwegian Research Centre (NORCE), Nygårdsporten 112, NO-5008, Bergen, Norway

## Abstract

• High heterogeneity and small size make nanoplastics a challenging issue.
• The One Health Approach is particularly relevant for nanoplastic research.
• Current limitations in nanoplastic research should not hinder mitigation strategies.
• Integrating nanoplastic data on humans, animals, and the environment is critical.

## Graphical abstract

**Figure:**
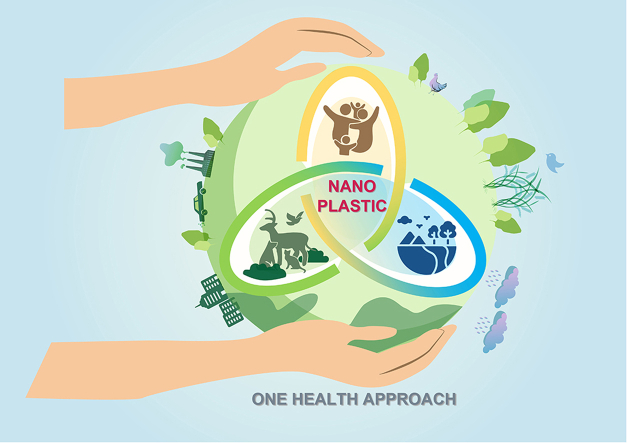

Large-scale production of plastics began in the 1950s followed by an onset of exponential growth, leading to its current widespread and dominant use. Despite this, no holistic risk assessment has yet been made on the health and environmental impacts of plastics, their degradation products, or their constituent chemicals. Once entering the environment, plastics undergo structural, chemical, and functional transformations, leading to the creation of microplastics and nanoplastics [1,2,3]. Environmental exposure to microplastics and nanoplastics becomes inevitable, causing direct human dietary exposure. A recent report of existing literature demonstrates human exposure via inhalation and ingestion, while pointing to concerns over the quality of studies [4]. The report further points to limited data on adverse effects in humans, while pinpointing nanoplastics as being of particular interest in terms of human health risk. The persistent nature of nanoplastics positions them as new “forever pollutants,” irreversibly accumulating in the environment and posing potential threats to both environmental and human health. Their high degree of heterogeneity, owing to the complex and dynamic mixture of polymers, additives, and other contaminants, makes nanoplastic pollution a highly challenging environmental issue.

In a timely review published by Dang et al. [5] in Eco-Environment & Health, the key knowledge gaps in nanoplastic research were identified and discussed as being (i) in exposure assessment and a mismatch between laboratory studies and real-world nanoplastic levels, (ii) a lack of knowledge on the nano-bio interactions including the underlying toxicological and pathological mechanisms of nanoplastic exposure, and (iii) consequences of interactions with other micro- and macro-molecules in the environment. This is the first publication calling for a comprehensive integrative scheme of a global “One Heath Approach” to produce robust multidisciplinary evidence of nanoplastic impacts on environmental and human health at the planetary level. In addition to the urgent need to fill the aforementioned scientific knowledge gaps, the authors also describe the need to address the entire plastic life cycle from plastic production, mishandled plastic waste, and incidental leakage into the environment, by giving play to the roles of different scientific communities, policymakers, and the public [6]. To date, quantitative data regarding the environmental exposure, ecotoxicological, and human health impacts of nanoplastics are rare due to technological limitations in sampling and detection [7] and the lack of precise exposure scenarios related to realistic scenarios [8]. However, it is vital that such limitations should not hinder the implementation of mitigation strategies. Unlike single chemical contaminants of emerging concern, an immediate ban on plastic production is impossible as plastics are firmly and systemically integrated into modern society, also described as the carbon lock-in of plastics [9]. This includes the use of plastics in industries and sectors such as building construction, regenerative energy production, transportation, healthcare instruments, and consumer goods. A reduction in plastic production is hampered by a lack of suitable strategies for safe and sustainable substitutive materials [10] as well as lobbyism in support of plastic-intensive consumer behavior [9]. Thus, reducing plastic production requires a full reconsideration of the consumerism of plastics in general. A better understanding of the exposure and effects of plastic pollution in general, including nanoplastics in particular, can support and guide future initiatives and actions.

As highlighted by Dang et al. [5], the hazards and risks of microplastics and nanoplastics remain uncertain. The map of global studies on microplastics and nanoplastics in food items, provided by Dang et al. [5], is a snapshot of available data on human exposure via the ingestion route. Such data poses questions regarding the comparability of current methods for sampling and analysis, while highlighting the unequal geographical distribution of scientific production on the topic. More realistic, higher-resolution, multiple-exposure-route maps of nanoplastic distribution and levels in different environmental matrices and organisms are needed for meaningful environmental and human risk assessments of nanoplastics. Also, from a clinical point of view, direct evidence of nanoplastics’ impacts on human health is severely lacking. For microplastics, some attempts have been made to monitor their presence in humans. As an indirect measure of plastic exposure, a study by the U.S. Centre of Disease Control (CDC) reported the presence of 13 different PET metabolites in the urine of 2,636 participants [11]. Integrating clinical data collection, tissue residue measurement, and causal analysis will pave the way to understanding the basis of the clinical effects of nanoplastics. For example, the involvement of human lung diseases, cardiovascular diseases, and cancer reports might be important to decipher the chronic impact of nanoplastics on human respiratory and cardiovascular systems [12]. In essence, continuing the progression of long-standing research in the field of ultrafine particles (defined as aerosols with aerodynamic diameter <100 nm) [13], one of the earliest studies detected plastic fibers in human lung tissues in 1998 [14]. Recently, more studies have detected microplastics in human lung samples [15,16]. Laboratory research efforts have also been made to reveal nanoplastic toxicity and underlying mechanisms with various human cell lines *in vitro* [17,18,19]. The underlying mechanisms of nanoplastic toxicity highly depend on the intrinsic properties that are particle-specific (e.g., size, surface charge, shape, and chemistry, including polymer type) [5,20]. However, a proper medical census of data is needed to fill the knowledge gap of the clinical effects of nanoplastics, requiring multidisciplinary studies involving medical doctors and environmental scientists.

We also agree with Dang et al. [5] that even a good understanding of the toxic effects and mechanisms of any single type of nanoplastics is insufficient to assess or predict nanoplastic toxicity as a whole. This is because environmental nanoplastics represent a complex mixture of plastic particles, organic/inorganic chemicals, and associated microorganisms that are then subject to numerous environmental transformations, affecting the nano-bio interface. For example, plastic particles can adsorb and concentrate toxic chemicals on their surface or interact with other biomolecules (forming protein corona) which can chemosensitize cells to toxic substances. Besides the unknown exposure levels of nanoplastics in the natural environment, many fundamental toxicodynamic and toxicokinetic questions on nanoplastic particles alone remain unanswered. For example, how do nanoplastic particles distribute into different cells, tissues, and organs? To what extent are nanoplastic particles excreted in different organisms? Are nanoplastic particles biomodified or biotransformed during translocation, circulation, or excretion? Are there specific molecular, cellular, tissue, or organ targets for nanoplastic particles? Also, ecotoxicological studies on nanoplastics still lack “real world” evidence on nanoplastic toxicity. Previous research on ultrafine particles may inform questions related to the inhalation of nanoplastics. Although it is recognized that nanoplastics represent a more complex contaminant suite than engineered nanomaterials [21], questions relating to the bioaccumulation dynamics and biological interactions of nanoplastics may be informed by the relatively rich data available on engineered nanomaterials [22].

Meaningful ecological and human health risk assessments of nanoplastics are only possible when we deeply and comprehensively understand their environmental fates, exposure levels, and systemic effects. Thus, achieving individual to systems-level assessments urgently needs a closer transdisciplinary and interdisciplinary collaboration approach, i.e., the concept of the “One Health Approach” [5]. Given the projected continuous and increasing production of plastic products and wastes [6], nanoplastics in the environment will only increase in the foreseeable future, accumulating in wildlife and human and potentially causing currently unknown biological effects and ecological impacts. It is time for more communities to consider and integrate the “One Health Approach,” in which polymer scientists, industry, environmental engineers, and policy-makers work together to design, produce, and remediate environmental plastics [5]. To alleviate and mitigate the risks of nanoplastic pollution, more determined actions are needed, including initiatives on regulations, legislation, and harmonization by international, national, and regional authorities in collaboration with other stakeholders [23,24,25].

As an issue initially raised by marine environmental scientists, microplastic and nanoplastic pollution has become a research priority and public concern, transitioning into concerns for human health. Conversely, research into ultra-fine particles and engineered nanomaterials took its starting point within the human health research community. Logically, any research area is initially influenced by its historical roots. However, it is important to acknowledge that a truly transdisciplinary and interdisciplinary collaborative research approach is needed to solve the nanoplastic problem. Mono-disciplinary studies on nanoplastic have been conducted, but whether and how nanoplastic pollution threatens the environment, health, and the economy has not been elucidated. It will require interdisciplinary dialog and collaboration on all possible levels to support and realize the holistic “One Health Approach.” Metaphorically, there is no silver bullet to the problem of nanoplastic pollution; we can only achieve “One Health” by taking into account the interlinks between humans, animals, plants, and their shared environments at the planetary level in future solutions and actions.

## Declaration of competing interest

The authors declare no conflicts of interest.
